# Successful hemostasis of spurting gastric ulcer bleeding using self-assembling peptide gel

**DOI:** 10.1055/a-2627-3791

**Published:** 2025-07-15

**Authors:** Masamichi Saegusa, Takehide Fukuchi, Shinpei Kondo, Shigeru Iwase, Shin Maeda

**Affiliations:** 1 36993 Department of Gastroenterology, Fujisawa City Hospital, Kanagawa, Japan; 2 26438 Department of Gastroenterology, Yokohama City University School of Medicine Graduate School of Medicine, Yokohama, Japan

Self-assembling peptide (SAP) hemostatic agents, such as a commercially available SAP gel (PuraStat; 3-D Matrix, Tokyo, Japan), have demonstrated efficacy in managing gastrointestinal bleeding, with high initial hemostasis rates and relatively low rebleeding rates 1 2 3 4 . In our recent report, we showed that this agent can be effective even when conventional devices are not applicable 5 . However, its role as a monotherapy for spurting arterial bleeding has not been established.

We present the first video-documented case of successful hemostasis using an SAP gel as monotherapy for a spurting gastric ulcer ( Video 1 ). A 63-year-old man presented with hematemesis. Emergency endoscopy revealed a Forrest Ia ulcer on the posterior wall of the upper stomach. Active spurting from an exposed vessel was observed, and hemostasis was initially attempted using coagulation forceps. However, this approach failed due to the tangential orientation of the ulcer and endoscope, which worsened the bleeding and impaired visibility.

Successful hemostasis of spurting gastric ulcer bleeding using self-assembling peptide gel.Video 1

In cases of spurting arterial bleeding ( Fig. 1 **a** ), close positioning of the endoscope can obscure visualization, and limited maneuverability may hinder the use of conventional tools such as forceps or clips ( Fig. 1 **b** ). In this case, an SAP gel was applied using a dedicated catheter from a non-contact, mid-distance position. This approach preserved endoscopic visibility and allowed for precise control ( Fig. 1 **c** ), even with respiratory motion. The transparent gel gradually controlled the bleeding, transitioning from spurting to pulsatile flow, until complete hemostasis was achieved within 3 minutes, without the need for additional intervention ( Fig. 1 **d, e** ).

**Fig. 1:**
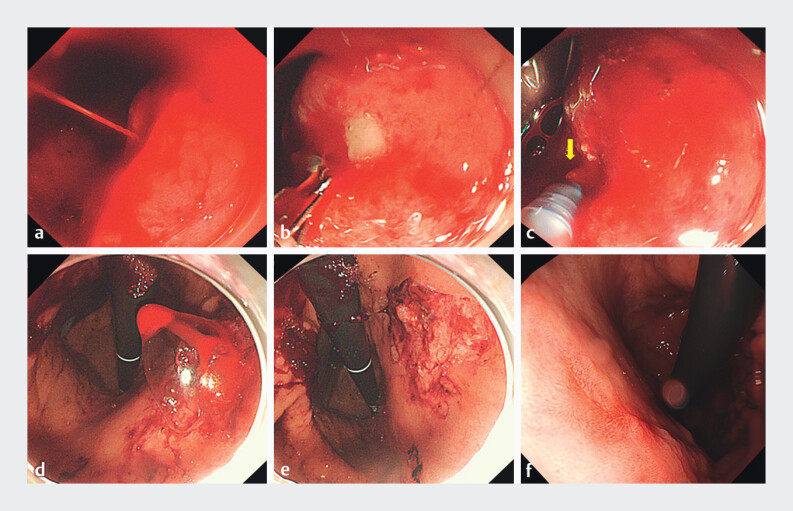
**a** Spurting arterial bleeding from a gastric ulcer located on the posterior wall of the upper gastric body. **b** Hemostasis attempted using hemostatic forceps. **c** Application of a self-assembling peptide (SAP) gel with identification of the bleeding point (yellow arrow). **d** The ulcer encapsulated by the gel, with effective bleeding control. **e** Complete hemostasis achieved. **f** Follow-up endoscopy at one month showing a healed ulcer with scarring.

Follow-up endoscopy 1 month later confirmed complete ulcer healing with no signs of rebleeding ( Fig. 1 **f** ). This case highlights the potential of SAP gel as an effective monotherapy for spurting arterial bleeding, particularly when conventional methods are difficult or infeasible.

Endoscopy_UCTN_Code_TTT_1AO_2AD

## Notes

Endoscopy E-Videos https://eref.thieme.de/e-videos
*E-Videos* is an open access online section of the journal *Endoscopy* , reporting on interesting cases and new techniques in gastroenterological endoscopy. All papers include a high-quality video and are published with a Creative Commons CC-BY license. Endoscopy E-Videos qualify for HINARI discounts and waivers and eligibility is automatically checked during the submission process. We grant 100% waivers to articles whose corresponding authors are based in Group A countries and 50% waivers to those who are based in Group B countries as classified by Research4Life (see: https://www.research4life.org/access/eligibility/ ). This section has its own submission website at https://mc.manuscriptcentral.com/e-videos .
